# Body mass index in adolescence, risk of type 2 diabetes and associated complications: A nationwide cohort study of men

**DOI:** 10.1016/j.eclinm.2022.101356

**Published:** 2022-03-21

**Authors:** Andréasson Karin, Edqvist Jon, Adiels Martin, Björck Lena, Lindgren Martin, Sattar Naveed, Lind Marcus, Åberg Maria, Rosengren Annika

**Affiliations:** a Department of Molecular and Clinical Medicine, Sahlgrenska Academy, University of Gothenburg, Diagnosvägen 11, Gothenburg 41650, Sweden; b Sahlgrenska University Hospital, Gothenburg, Sweden; c Biostatistics, School of Public Health and Community Medicine, Institute of Medicine, Sahlgrenska Academy, University of Gothenburg, Gothenburg, Sweden; d Region Västra Götaland, Sahlgrenska University Hospital, Department of Medicine Geriatrics and Emergency Medicine, Östra, Gothenburg, Sweden; e Institute of Cardiovascular and Medical Sciences, University of Glasgow, Glasgow, UK; f Department of Medicine, NU-Hospital Group, Uddevalla, Sweden; g School of Public Health and Community Medicine, Primary Health Care, Institute of Medicine, Sahlgrenska Academy, University of Gothenburg, Sweden; h Region Västra Götaland, Regionhälsan, Gothenburg, Sweden; i Department of Cardiology, Sahlgrenska University Hospital, Diagnosvägen 11,Östra, Gothenburg 41650, Sweden

**Keywords:** type 2 diabetes, obesity

## Abstract

**Background:**

Obesity is a predominant factor in development of type 2 diabetes but to which extent adolescent obesity influences adult diabetes is unclear. We investigated the association between body mass index (BMI) in young men and subsequent type 2 diabetes and how, in diagnosed diabetes, adolescent BMI relates to glycemic control and diabetes complications.

**Methods:**

Baseline data from the Swedish Conscript Register for men drafted 1968–2005 was combined with data from the National Diabetes and Patient registries. Diabetes risk was estimated through Cox regression and Kaplan-Meier survival estimates. Relationships between BMI, glycemic control and diabetes complications were assessed through multiple linear and logistic regression.

**Findings:**

Among 1,647,826 men, 63,957 (3·88%) developed type 2 diabetes over a median follow-up of 29.0 years (IQR[21.0–37.0]). The risk of diabetes within 40 years after conscription was nearly 40% in individuals with adolescent BMI ≥35 kg/m^2^. Compared to BMI 18·5–<20 kg/m^2^ (reference), diabetes risk increased in a linear fashion from HR 1·18(95%CI 1·15–1·21) for BMI 20–<22·5 kg/m^2^ to HR 15·93(95%CI 14·88–17·05) for BMI ≥35 kg/m^2^, and a difference in age at onset of 11·4 years was seen. Among men who developed diabetes, higher adolescent BMI was associated with higher HbA1c levels and albuminuria rates.

**Interpretation:**

Rising adolescent BMI was associated with increased risk of type 2 diabetes diagnosed at a younger age, with poorer metabolic control, and a greater prevalence of albuminuria, all suggestive of worse prognosis.

**Research in context**

**Evidence before this study**

We searched PubMed for publications without language restriction, published before 2021-10-18, using search terms body mass index, BMI, overweight, obesity or body weight and adolescence, youth or teenager, and diabetes mellitus type 2 or type 2 diabetes with search terms found in abstract, title or MESH headings, further restricting to publications with diabetes in the title. Additional publications were found using references of articles found in the database search.

Elevated BMI is the predominant risk factor for type 2 diabetes, but less is known regarding how BMI at different life stages affects diabetes risk. In childhood and adolescence, high BMI is associated with elevated risk of type 2 diabetes, although attenuated if BMI is normalized during adulthood. Studies have also shown an association between the duration of obesity over the life course and diabetes risk. In individuals who develop diabetes, overweight and obesity, as well as early disease onset, is associated with worse glycemic control, and with higher risk of diabetes complications, cardiovascular disease and mortality.

Although previous studies have shown an association between adolescent body weight and type 2 diabetes, findings have been heterogenous, influenced by study size, method of assessment and definition of diabetes diagnosis. Few studies have been powered to quantify diabetes risk among the severely obese. Little is known regarding how BMI in adolescence relates to the prognosis of type 2 diabetes regarding metabolic control and diabetes complications.

**Added value of this study**

High adolescent BMI was strongly associated with incident type 2 diabetes with an increased risk seen already at BMI levels considered normal. Hazard ratios (HRs) increased in a linear fashion from HR 1·18 (95% CI 1·15–1·21) for BMI 20–< 22·5 kg/m^2^ to HR 15·93 (95% CI 14·88–17·05) for BMI ≥ 35 kg/m^2^ with BMI 18·5–< 20 kg/m^2^ as reference. Men with high adolescent BMI developed type 2 diabetes at a far younger age, with poorer metabolic control, higher blood pressure, LDL cholesterol and albuminuria rates at diabetes onset.

**Implications of all the available evidence**

Being overweight or obese in adolescence is strongly associated with an increased risk of type 2 diabetes later in life. The high and increasing prevalence of obesity in the young will likely contribute to a continued rise in type 2 diabetes, being diagnosed in younger patients, with poorer metabolic control at onset and a greater risk of complications. The available results indicate a dire need for prevention of overweight and obesity in society, initiated at an early age.

**Search terms in full**

(body mass index[mh] OR "body mass index"[tiab] OR BMI[tiab] OR overweight[mh] OR overweight[tiab] OR obesity[mh] OR obesity[tiab] OR body weight[mh] OR "body weight"[tiab]) AND (Adolescence[mh] OR youth[tiab] OR teenager[tiab] OR teenagers[tiab] OR adolescent[tiab] OR adolescents[tiab] OR adolescence[tiab]) AND (Diabetes mellitus type 2[mh] OR "Diabetes mellitus type 2″[tiab] OR "Diabetes type 2″[tiab] OR "type 2 diabetes"[tiab]) AND (diabetes[ti])

**End publication date 2021-10-18, no restriction of start date**

Search initiation 2021-10-18 lead to 2741 results.

Alt-text: Unlabelled box

## Introduction

Overweight and obesity, where rising rates worldwide are strikingly evident in the young,^1^ is associated with several types of cancer, cardiovascular disease and type 2 diabetes.^2^ In 2011, 366 million people globally were estimated to have diabetes, a number expected to reach 552 million by 2030.^3^ Obesity is a predominant factor in the development of type 2 diabetes, but with previous studies showing marked heterogeneity in relative risk.^4^

An association has been shown between the duration of obesity as well as weight gain during puberty and risk of type 2 diabetes, indicating early-onset obesity as an important risk factor,^5^^,^^6^ although attenuated when adjusting for adult BMI.^7^^,^^8^ Studies on Israeli conscripts have shown a positive association between BMI and future risk of type 2 diabetes,^8^, ^9^, ^10^, ^11^ however, with imprecise estimates, due to a low prevalence of obesity, in particular of severe obesity. Previous studies also vary in how a diagnosis of diabetes was established, either self-reported,^7^ by using information from patient registers,^12^^,^^13^ or as diabetes mortality.^9^ As many patients with type 2 diabetes are managed in primary care only,^14^ this may lead to an underestimated number of cases.

Type 2 diabetes is associated with risk of microvascular complications, mortality and cardiovascular events, where low age at diagnosis, poor glycemic control and presence of cardiovascular risk factors are associated with poorer prognosis.^15^, ^16^, ^17^, ^18^, ^19^ The effect of obesity in adolescence on metabolic control at diagnosis of type 2 diabetes has, to our knowledge, not been extensively investigated. Using data from a national large cohort of male conscripts, linked to the Swedish National Diabetes Register (NDR) with detailed data on almost 90% of diabetes cases in Sweden^20^^,^^21^ we sought first, to investigate the association between BMI in adolescence and the risk of subsequent type 2 diabetes and second, to assess the association between early BMI and risk factor control - metabolic control, blood pressure, blood lipids, and microalbuminuria – among those who were diagnosed with type 2 diabetes during their adulthood. The high coverage of the NDR, at present estimated at almost 90% of diabetes cases in Sweden, and the detailed data,^20^^,^^21^ allow not only for a near-complete capture of cases, but also for investigation of risk factor pattern post-onset of diabetes type 2.

## Methods

### Study design and participants

Between 1901 and 2010 all Swedish men were obligated by law to enlist for military service, with exceptions only for men with severe physical or mental disorders, disabilities or for those serving time in prison. The conscripts underwent a two-day sequence of standardized tests evaluating physical strength, cardiorespiratory fitness and intelligence, and were seen by a physician and a psychologist, with medical history and pre-existing morbidities recorded using International classification of disease (ICD) codes. Height was measured using wall-mounted stadiometers and weight was measured using analogue or digital scales. Height was rounded to the nearest cm, and weight to the nearest kg. Blood pressure was measured at rest in the sitting position. Cardiorespiratory fitness was evaluated by bicycle ergometry, where maximum work capacity (W) was measured and divided by body weight, and muscle strength was measured through isometric testing of maximal knee extension, elbow flexion and handgrip strength. Cognitive performance was evaluated through logical, verbal, visuospatial and technical cognition. Scores were transformed into stanine scores.^22^, ^23^, ^24^

Data were entered in digital form from 1968. The study cohort comprised men born in 1950 to 1987, enlisting in 1968 (start of follow-up) to 2005 (*n* = 1874651). Individuals were excluded if enlisting early (age <18 years) or late (age >24 years) (*n* = 60,113), if BMI was missing at conscription (*n* = 149,099), or if diabetes onset was before or during the same year as conscription (*n* = 1301) **(Supplementary Figure S1).** From the remaining 1664138, individuals were excluded if follow-up time was negative (presumed incorrect entry) (*n* = 170), if height was <140 cm or >210 cm or if weight was <50 kg or >140 kg (*n* = 9485). Individuals were also excluded if there was a record at baseline of alcohol or substance abuse, cancer, congenital heart disease, or any cardiovascular disease (*n* = 6657). Individuals were followed until diabetes diagnosis, death, or until December 31, 2016.

The NDR, initiated in 1996, is a tool for quality assurance in diabetes care. In 2017, 425,860 patients were registered in the NDR, covering almost 90% of persons with diabetes in Sweden.^20^^,^^21^^,^^16^ All individuals provide informed consent. As the NDR captures prevalent cases the majority of men with diabetes type 2 with onset at a younger age would have been registered during the early years of the NDR (with the exception of a probably very minor proportion of men with early diabetes who died or emigrated between onset and 1997). The register is updated annually via electronic clinical records or by data registered online, with information on diabetes type, year of diagnosis, treatment, diabetes complications, and risk factors. Diabetes duration in the NDR is estimated as the time (years) between the first recorded diagnosis of diabetes and the time of registration. HbA1c is measured as mono-S converted into mmol/mol (IFCC), blood lipids are measured in mmol/L, blood pressure in mmHg, and BMI is calculated through weight in kilograms divided by meters squared. Albuminuria is defined as two out of three positive urine samples taken within a year, where a U-albumin of 20–200 µg/min or 20–300 mg/L or an albumin/creatinine ratio 3–30 mg/mmol is defined as microalbuminuria and a U-albumin of >200 µg/min or >300 mg/L, or albumin/creatinine ratio >30 mg/mmol is defined as macroalbuminuria.

The Swedish NPR (National patient registry) starting inclusion of patient records from hospitalizations successively from 1970, is near-complete since 1987 (inpatient register), and includes specialist outpatient visits (outpatient register) since 2001. A validation of the inpatient register diagnoses has shown a positive predictive value (PPV) of 85–95% for most major cardiovascular diagnoses.^25^

### Procedures

The definitions of type 2 diabetes and age of onset used are shown in **Supplementary Table S1**. For the purpose of this study, we primarily used the clinical definition of type 2 diabetes registered in the NDR by the physician in charge of the patient. Year of diabetes onset was taken from registration in the NDR, with the most commonly entered value used in case of inconsistency. If year of onset according to the NDR was later than the year of first registration (presumed incorrect entry), registration year was used. If an individual was registered in the NDR but year of onset was missing, the first year of registration in the NPR containing an ICD code of 250 (ICD-9 or ICD-8) or E11 (ICD-10) was used. If no year of onset was recorded and no ICD-code of diabetes was registered, registration year in the NDR was used as year of onset. Subjects with other diabetes types registered in the NDR, missing clinical definition, or with diabetes registered in the NPR only (i.e., not registered in the NDR), were censored at the year of onset (NDR) or year of diagnosis (NPR).

In a sensitivity analysis, we further included conscripts registered in the NPR with type 2 diabetes using the same definition as above, but who were not registered in the NDR **(Supplementary Table S1).** With an existing ICD-10 code of E11 at any time during follow-up, the year of first registered diabetes ICD-code was set as year of onset, independent of which specific type of diabetes diagnosis that occurred as the first registered. If no ICD-10 code of E11 was registered, other ICD 8-, 9- or 10 code for diabetes was used with the additional criterium of onset after 30 years of age, as ICD-8 and 9 did not specify diabetes type. Individuals with ICD-10 code of E10 (type 1 diabetes) were censored at diagnosis.

The study was approved by the regional ethics committee of the University of Gothenburg.

### Statistical analyses

Based on prior findings of risk of cardiovascular disease in this cohort^24^ we stratified our study cohort into categories by BMI at time of conscription: <18·5; 18·5–<20; 20–<22·5; 22·5–<25; 25–<27·5; 27·5–<30; 30–<32·5; 32·5–<35; ≥35 kg/m^2^. With respect to risk of future type 2 diabetes, Kaplan-Meier survival estimates were calculated. We also performed Cox regression analyses with reference level set to BMI 18·5–<20 kg/m^2^, where model 1 was adjusted for age, year of conscription and conscription center. Model 2 was additionally adjusted for cardiorespiratory fitness and muscle strength. In a supplementary analysis we further adjusted for parental education. In a separate supplementary analysis, results were stratified by muscle strength, cardiorespiratory fitness and IQ. Individuals were followed until diabetes diagnosis, death, or until December 31, 2016.

In order to analyze the level of risk factor control in terms of HbA1c, systolic blood pressure (SBP), albuminuria and LDL cholesterol with regard to baseline BMI, we used multiple linear regression (continuous outcomes) with least square means and logistic regression (binary outcomes) adjusted for age and diabetes duration using CI 95% and BMI groups <18·5; 18·5–<25; 25–<30; 30–<35 and ≥35 kg/m^2^, with the purpose to increase the power of the estimates. We further performed a sensitivity analysis additionally adjusting for weight change between the time of conscription and first entry in NDR.

To visualize the patterns of weight change for individuals who developed diabetes, we created a river plot, using BMI groups <18·5; 18·5 - 25; 25–<30; 30–<35 and ≥35 kg/m^2^ in order to increase interpretability compared to BMI groups consisting of only 2·5 kg/m^2^ steps.

### Role of the funding source

The funders of the study had no role in study design, data collection, data analysis, data interpretation, or writing of the report. All authors had full access to all the data in the study and the corresponding author had final responsibility for the decision to submit for publication.

## Results

### Study participants at conscription

After exclusions (**SupplementaryFigure S1**), 1,647,826 men with a mean age of 18·3 years remained for analysis. Table 1 shows baseline characteristics by BMI at conscription. At conscription, 12·2% (*n* = 201,828) had BMI ≥25 kg/m^2^, including 2·22% (*n* = 36,616) with BMI ≥30 kg/m^2^. Conscripts with higher BMI had higher mean systolic and diastolic blood pressure, greater muscle strength, lower IQ, lower cardiorespiratory fitness and a later year of conscription compared to leaner men.

**Table 1:** Baseline characteristics for individuals at conscription, stratified by body mass index (kg/m^2^) at conscription.

	Overall	<18.5	18.5–<20	20–<22.5	22.5–<25	25–<27.5	27.5–<30	30–<32.5	32.5–<35	35 or above
*n*	1,647,826	126,802	298,195	668,832	352,169	122,079	43,133	20,439	9395	6782
Age	18.3 (0.6)	18.3 (0.6)	18.3 (0.6)	18.3 (0.6)	18.3 (0.6)	18.3 (0.7)	18.3 (0.7)	18.3 (0.6)	18.3 (0.7)	18.3 (0.7)
Median year of conscription	1986 [1977,1994]	1983 [1975,1991]	1984 [1976,1992]	1986 [1977,1993]	1988 [1980,1995]	1989 [1981,1996]	1990 [1982,1998]	1992 [1983,1999]	1993 [1985,2000]	1995 [1987,2001]
Body mass index (kg/m2)	21.9 (3.0)	17.7 (0.6)	19.3 (0.4)	21.2 (0.7)	23.5 (0.7)	26.1 (0.7)	28.6 (0.7)	31.1 (0.7)	33.6 (0.7)	37.4 (2.1)
Systolic blood pressure (mmHg)	128.5 (10.9)	125.6 (10.8)	126.7 (10.8)	128.2 (10.8)	129.9 (10.8)	131.3 (10.8)	132.5 (11.1)	133.6 (11.0)	134.7 (11.2)	136.2 (11.4)
Diastolic blood pressure (mmHg)	67.6 (9.8)	67.4 (9.6)	67.2 (9.6)	67.3 (9.7)	67.7 (9.9)	68.4 (10.0)	69.2 (10.3)	70.0 (10.5)	71.4 (10.7)	72.7 (11.1)
Parental education (highest achieved)										
Low (1–2)	416,734 (26.1)	35,594 (29.2)	78,885 (27.5)	165,694 (25.6)	85,015 (24.9)	30,869 (26.1)	11,285 (26.9)	5264 (26.5)	2427 (26.6)	1701 (25.7)
Medium (3–5)	702,350 (44.1)	52,370 (43.0)	123,068 (42.8)	279,754 (43.2)	152,864 (44.7)	55,512 (46.9)	20,543 (49.0)	9981 (50.3)	4712 (51.6)	3546 (53.5)
High (6–7)	474,806 (29.8)	33,838 (27.8)	85,255 (29.7)	201,702 (31.2)	103,888 (30.4)	32,075 (27.1)	10,062 (24.0)	4610 (23.2)	1994 (21.8)	1382 (20.8)
Muscle strength										
Low (1–3)	220,848 (13.5)	50,986 (41.0)	64,488 (21.7)	70,245 (10.6)	22,203 (6.3)	7597 (6.3)	2856 (6.7)	1402 (7.0)	594 (6.8)	477 (8.5)
Medium (4–6)	928,899 (56.8)	69,680 (56.0)	200,763 (67.7)	410,451 (61.7)	167,201 (47.7)	50,813 (41.9)	17,082 (40.0)	7636 (38.2)	3229 (36.7)	2044 (36.5)
High (7–9)	485,719 (29.7)	3736 (3.0)	31,446 (10.6)	185,060 (27.8)	160,872 (45.9)	62,815 (51.8)	22,779 (53.3)	10,961 (54.8)	4971 (56.5)	3079 (55.0)
Cardiorespiratory fitness										
Low (1–4)	246,372 (15.4)	37,597 (30.9)	51,599 (17.7)	66,843 (10.2)	34,900 (10.2)	22,786 (19.3)	14,406 (34.8)	9639 (50.5)	5001 (61.3)	3601 (73.3)
Medium (5–6)	782,307 (48.8)	69,550 (57.1)	167,118 (57.2)	315,636 (48.2)	147,971 (43.1)	54,782 (46.4)	17,806 (43.0)	6453 (33.8)	2146 (26.3)	845 (17.2)
High (7–9)	575,980 (35.9)	14,659 (12.0)	73,418 (25.1)	272,939 (41.6)	160,755 (46.8)	40,555 (34.3)	9169 (22.2)	2999 (15.7)	1016 (12.4)	470 (9.6)
Global IQ										
Low (1–3)	326,475 (20.0)	26,812 (21.4)	58,440 (19.7)	121,136 (18.2)	68,428 (19.6)	28,791 (23.8)	11,925 (27.9)	5958 (29.7)	2865 (31.9)	2120 (34.8)
Medium (4–6)	896,730 (54.8)	65,720 (52.5)	158,495 (53.5)	364,835 (54.9)	196,668 (56.2)	68,234 (56.4)	23,609 (55.3)	11,052 (55.1)	4895 (54.5)	3222 (52.9)
High (7–9)	411,745 (25.2)	32,612 (26.1)	79,405 (26.8)	178,992 (26.9)	84,606 (24.2)	23,979 (19.8)	7138 (16.7)	3043 (15.2)	1219 (13.6)	751 (12.3)
Hypertension	1330 (0.1)	49 (0.0)	119 (0.0)	354 (0.1)	283 (0.1)	182 (0.1)	127 (0.3)	85 (0.4)	55 (0.6)	76 (1.1)

Data as n (%) for categorical variables, mean (sd) for continuous variables and median [IQR] for year of conscription.

Parental education defined as 1: < 9 years, 2: pre- high school education of 9 years, 3: high school education, 4: university < 2 years, 5: university ≥ 2 years, 6: post graduate education, 7: postgraduate research training. Muscle strength, Cardiorespiratory fitness and Global IQ represented as stanine scores.

**Figure 1:**
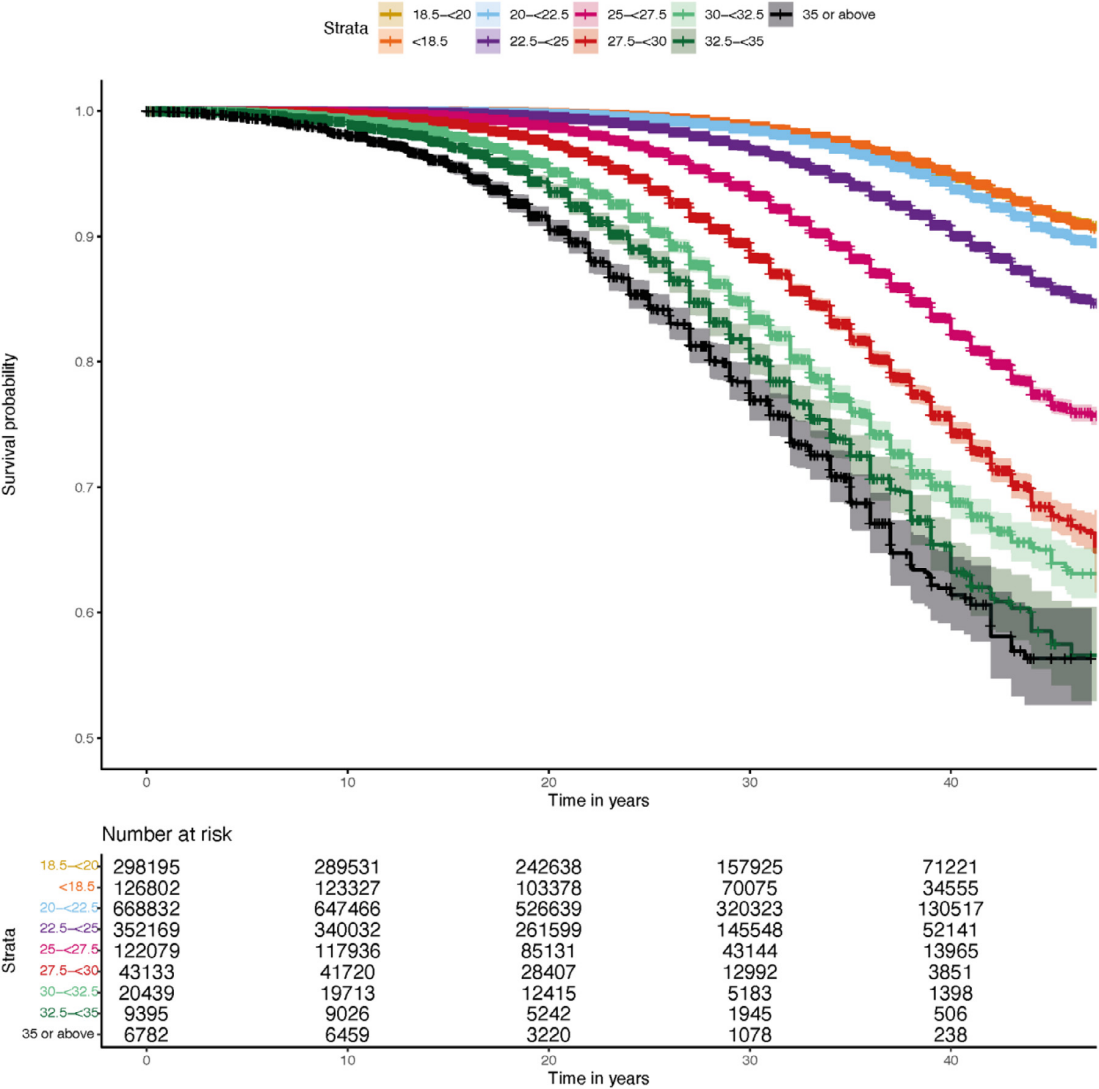
**Kaplan Meier Survival curve for incident type 2 diabetes stratified by body mass index at conscription.** Kaplan Meier survival estimates. Individuals at risk by each 10-year period are presented at the bottom of the figure. Regions around lines denote 95% CI. Abbreviations: body mass index (BMI). Log rank test p-value: 2 × 10^−16^.

**Figure 2:**
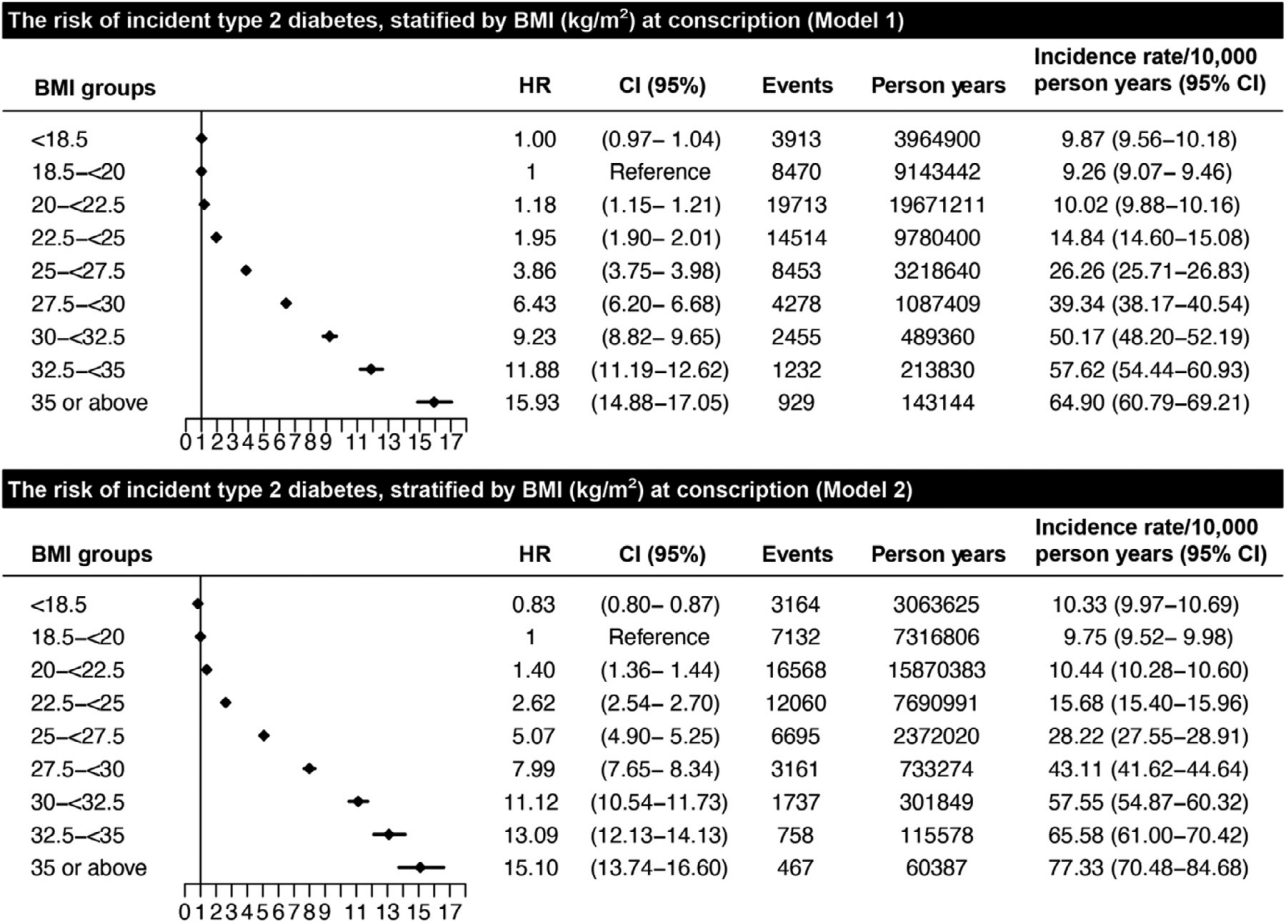
**Hazard ratio and incidence rates for the risk of incident type 2 diabetes during follow- up, stratified by body mass index at conscription.** Analyses were based on Cox regression. Model 1, adjusted for age, year of conscription and center. Model 2, adjusted for age, year of conscription and center, cardiorespiratory fitness, and muscle strength. Rate as events per 10,000 person years. Abbreviations: body mass index (BMI).

Survival curves based on Kaplan Meier estimates showing risk of type 2 diabetes stratified by BMI at conscription are presented in Figure 1. 63,957 men developed type 2 diabetes over a median follow-up of 29·0 (IQR[21,0–27,0]) years. Estimated risk of type 2 diabetes 40 years after conscription was almost 40% in individuals with adolescent BMI ≥35 kg/m^2^ compared to less than 10% in individuals with BMI <18·5 kg/m^2^. Figure 2 shows crude incidence rates (per 10,000 person-years) and hazard ratios for type 2 diabetes with 95% CIs, stratified by BMI at conscription, with BMI 18·5–<20 kg/m^2^ as reference. Model 1 was adjusted for age, year of conscription and conscription center, and model 2 additionally adjusted for cardiorespiratory fitness and muscle strength. A stepwise risk increase for future type 2 diabetes was seen with increasing BMI at conscription in both models, with HR 1·18 (CI 95% 1·15–1·21) at BMI 20–<22·5 kg/m^2^ in model 1, and HR 1·40 (CI 95% 1·36–1·44) in model 2. The group with the highest BMI (≥35 kg/m^2^) had HR 15·93 (CI 95% 14·88–17·05) in model 1 and 15·10 (CI 95% 13·74–16·60) in model 2. **Supplementary Figure S5** shows a sensitivity analysis additionally controlled for parental education, which did not significantly alter the results.

Group characteristics at first registration in the NDR for the 63,957 men with a registered diagnosis of type 2 diabetes at any time during follow-up, stratified by BMI at conscription are shown in Table 2. Mean diabetes duration before registration in the NDR was 2·2 (SD ± 3·9) years. Two thirds (66·8%) of the men with subsequent diabetes had adolescent BMI within the normal range (BMI 18·5–<25·0 kg/m^2^). Overall, 20·4% of subjects were smokers, with a slightly larger proportion of smokers (23·8%) in subjects with BMI ≥35 kg/m^2^. Men with low normal adolescent BMI (20·0–<22·5 kg/m^2^) were on average 52·8 (SD± 6·7) years old when registered in the NDR with a mean diabetes duration of 2·0 (SD± 3·7) years, whereas those with BMI ≥35 kg/m^2^ were 41·4 (SD± 8·8) years old with mean diabetes duration 3·0 (SD± 4·7) years. Men in the latter category also had higher BMI at NDR registration, with a mean BMI of 41·3 (SD ± 7·1) kg/m^2^ while those with adolescent BMI of 18·5–<20 kg/m^2^ had mean BMI 28·8 (SD ± 4·2) kg/m^2^.

**Table 2:** . Characteristics at registration in the NDR, stratified by body mass index (kg/m^2^) at conscription.

	Missing data n (%)	Overall	<18.5	18.5–<20	20–<22.5	22.5–<25	25–<27.5	27.5–<30	30–<32.5	32.5–<35	35 or above
n		63957	3913	8470	19713	14514	8453	4278	2455	1232	929
Age	0 (0)	51.2 (7.5)	54.2 (6.4)	53.6 (6.5)	52.8 (6.7)	51.1 (7.1)	49.3 (7.4)	47.5 (7.8)	45.4 (8.0)	44.1 (8.5)	41.4 (8.8)
Diabetes duration	3650 (5.7)	2.2 (3.9)	2.0 (3.7)	1.9 (3.5)	2.0 (3.7)	2.2 (3.8)	2.4 (4.1)	2.6 (4.2)	2.9 (4.7)	2.9 (4.6)	3.0 (4.7)
Body mass index (kg/m2)	15664 (24.5)	32.0 (5.7)	27.4 (3.9)	28.8 (4.2)	30.4 (4.5)	32.5 (4.9)	34.2 (5.2)	35.9 (5.6)	37.2 (5.9)	39.0 (6.5)	41.3 (7.1)
HbA1c (mmol/mole)	6972 (10.9)	58.0 (19.5)	56.3 (19.0)	56.1 (18.5)	56.7 (18.8)	58.2 (19.5)	59.6 (20.1)	60.3 (20.0)	62.1 (20.9)	62.2 (20.9)	64.2 (22.2)
Systolic blood pressure (mmHg)	9591 (15)	135.9 (15.9)	135.2 (16.0)	135.4 (16.0)	135.5 (15.8)	136.0 (15.9)	136.6 (15.8)	136.9 (16.0)	137.1 (15.6)	138.3 (16.8)	137.7 (16.6)
Diastolic blood pressure (mmHg)	9661 (15.1)	83.3 (10.1)	82.4 (9.7)	82.7 (9.9)	82.9 (9.9)	83.6 (10.1)	83.9 (10.1)	83.9 (10.2)	84.1 (10.4)	84.3 (10.7)	84.1 (11.0)
Total cholesterol (mmol/L)	20218 (31.6)	5.2 (1.3)	5.2 (1.2)	5.2 (1.3)	5.2 (1.3)	5.2 (1.2)	5.1 (1.3)	5.0 (1.2)	5.0 (1.2)	5.0 (1.1)	5.0 (1.3)
LDL cholesterol (mmol/L)	25624 (40.1)	3.1 (1.0)	3.1 (1.0)	3.1 (1.0)	3.1 (1.0)	3.1 (1.0)	3.0 (1.0)	2.9 (1.0)	2.9 (0.9)	2.9 (0.9)	2.9 (0.9)
HDL_cholesterol	24127 (37.7)	1.1 (0.3)	1.2 (0.4)	1.2 (0.3)	1.1 (0.3)	1.1 (0.3)	1.1 (0.3)	1.1 (0.3)	1.0 (0.3)	1.0 (0.3)	1.0 (0.4)
TC/HDL-c	24388 (38.1)	5.0 (1.8)	4.8 (1.6)	4.9 (1.7)	4.9 (1.7)	5.0 (1.8)	5.1 (2.1)	5.1 (2.2)	5.2 (1.9)	5.0 (1.7)	5.2 (1.8)
Albuminuria	33289 (52)										
No albuminuria		25720 (83.9)	1622 (87.8)	3487 (85.9)	8034 (85.5)	5790 (83.5)	3386 (81.6)	1688 (81.3)	928 (78.8)	440 (75.7)	345 (77.0)
Mikroalbuminuria		4591 (15.0)	215 (11.6)	529 (13.0)	1280 (13.6)	1073 (15.5)	696 (16.8)	357 (17.2)	226 (19.2)	124 (21.3)	91 (20.3)
Macroalbuminuria		357 (1.2)	11 (0.6)	43 (1.1)	84 (0.9)	70 (1.0)	65 (1.6)	31 (1.5)	24 (2.0)	17 (2.9)	12 (2.7)
Smokers	14802 (23.1)	10014 (20.4)	610 (20.5)	1357 (21.1)	3058 (20.2)	2140 (19.1)	1354 (20.7)	703 (21.4)	403 (20.8)	219 (22.8)	170 (23.8)
Statins	5905 (9.2)	22088 (38.0)	1389 (39.2)	3089 (40.1)	7079 (39.4)	5036 (38.2)	2800 (36.6)	1413 (36.5)	707 (32.0)	346 (30.9)	229 (27.2)
Antihypertensives	5801 (9.1)	31742 (54.6)	1905 (53.7)	4083 (53.2)	9720 (54.3)	7297 (55.2)	4306 (55.8)	2172 (56.0)	1209 (53.9)	623 (55.1)	427 (50.3)
Antidiabetic treatment	2473 (3.9)										
No medication		20086 (32.7)	1400 (37.5)	3048 (37.6)	6617 (34.9)	4485 (32.1)	2375 (29.2)	1111 (26.9)	573 (24.2)	273 (22.9)	204 (22.8)
Oral agents		31668 (51.5)	1766 (47.3)	3883 (47.9)	9626 (50.8)	7261 (52.0)	4371 (53.7)	2257 (54.7)	1341 (56.6)	655 (54.9)	508 (56.8)
Insulin only		3845 (6.3)	287 (7.7)	581 (7.2)	1167 (6.2)	789 (5.7)	508 (6.2)	243 (5.9)	148 (6.2)	72 (6.0)	50 (5.6)
Oral agents/Insulin		5486 (8.9)	265 (7.1)	564 (7.0)	1444 (7.6)	1321 (9.5)	834 (10.2)	471 (11.4)	287 (12.1)	176 (14.8)	124 (13.9)
GLP-1		399 (0.6)	16 (0.4)	33 (0.4)	100 (0.5)	99 (0.7)	58 (0.7)	46 (1.1)	22 (0.9)	16 (1.3)	9 (1.0)

Data as n (%) for categorical variables and mean (sd) for continuous variables.

**Figure 3:**
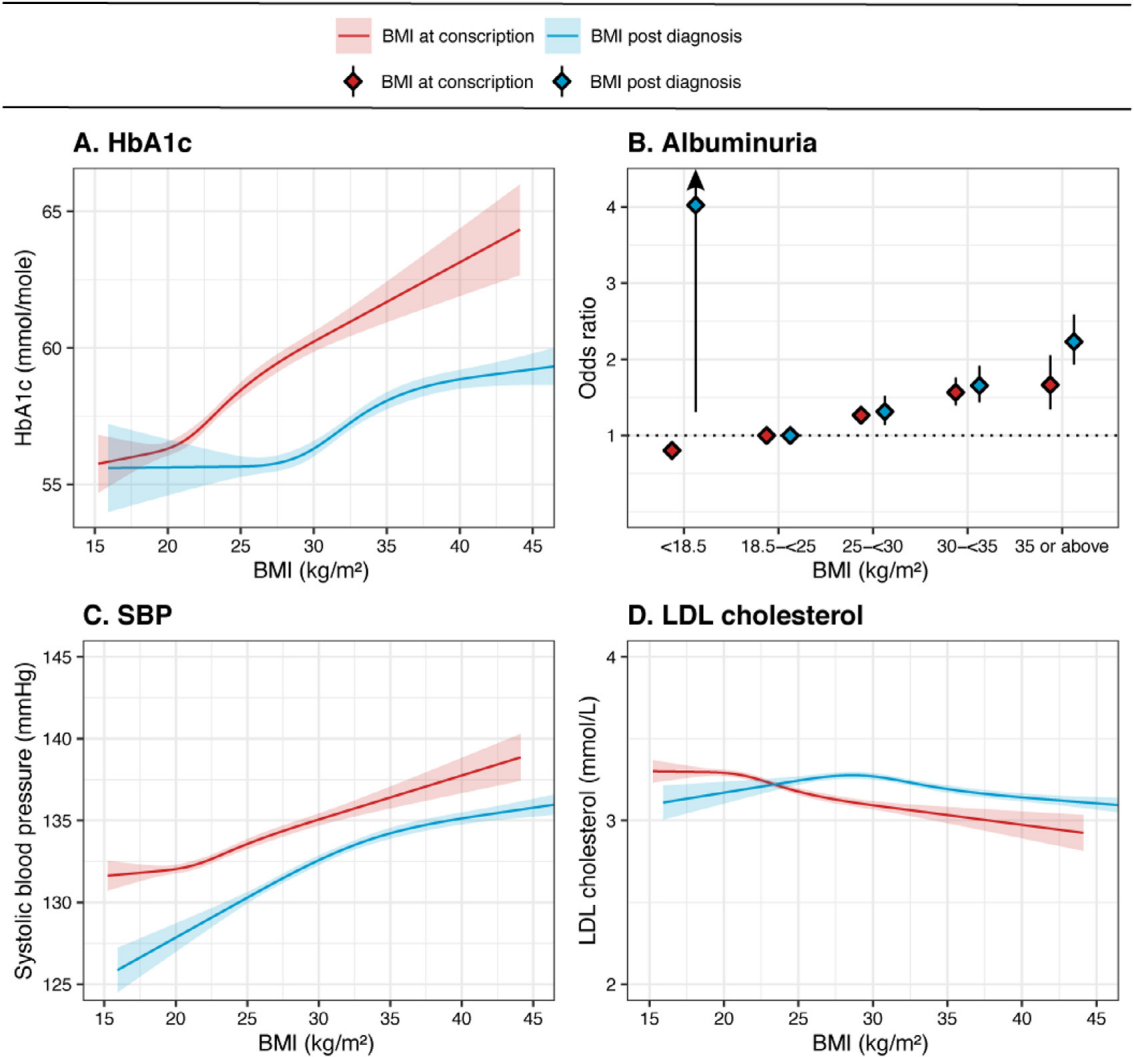
**Mean adjusted values for continuous risk factors and odds ratio for albuminuria post diagnosis for type 2 diabetes predicted by BMI at conscription and by BMI at first registration in NDR.** Linear Model Estimation using Ordinary Least Square (panels A, C, D) and logistic regression (panel B). All models adjusted for age and diabetes duration. BMI tied with 4 equal knots. Abbreviations: body mass index (BMI). Systolic blood pressure adjusted for use of antihypertensive medication. LDL adjusted for use of statins. Number of patients missing due to missing data (outcome or covariates): Panel *A* = 9809; Panel *B* = 33,365; Panel C = 12,358, Panel *D* = 27,593.

The results of risk factor analyses are shown in Figure 3, with mean values of HbA1c, SBP and LDL cholesterol based on linear regression and ordinary least square means, and odds ratio for albuminuria based on logistic regression, all adjusted for age and diabetes duration, predicted by (1) BMI at conscription and (2) BMI at NDR registration. SBP was adjusted for use of antihypertensive medication and LDL cholesterol was adjusted for use of statins. Elevated BMI at time of conscription was associated with higher levels of HbA1c, SBP and probability of albuminuria, but no association was seen with LDL cholesterol. When instead investigating associations for BMI post diagnosis, there was a considerably flatter association with HbA1c. Underweight was associated with increased risk of albuminuria, although the subgroup of individuals with BMI <18·5 kg/m^2^ at NDR registration consisted of only 15 individuals.

**Figure 4:**
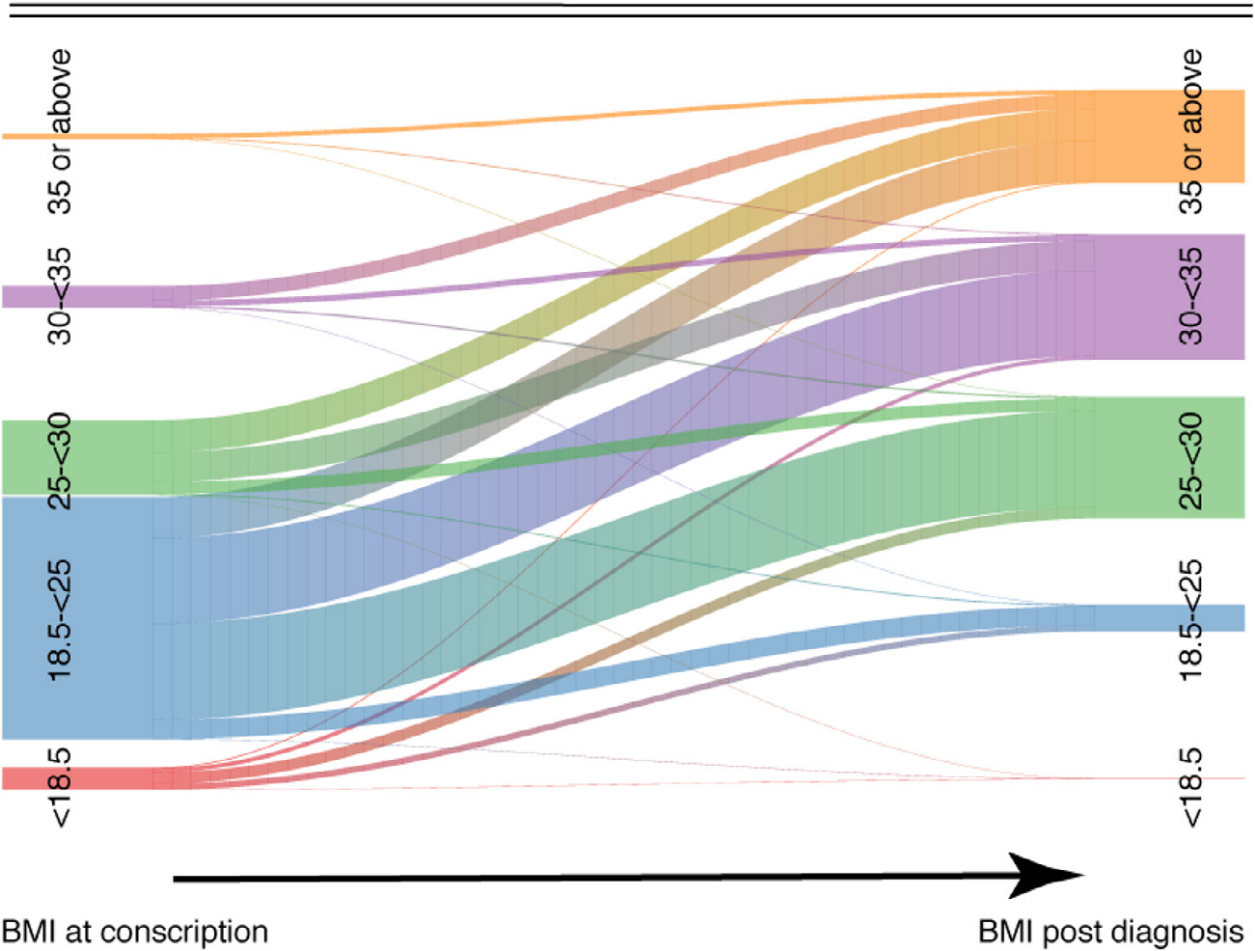
**Riverplot decribing patterns of weight change among indivuals who were diagnosed with type 2 diabetes between the time of conscription and post diagnosis (registration in the NDR).** Abbreviations: body mass index (BMI). Thickness of the lines represent the number of participants at the corresponding level of BMI.

Figure 4 and **supplementary Table S2** show BMI change between time of conscription and after diagnosis of type 2 diabetes. The majority of those registered in the NDR experienced a substantial weight gain regardless of BMI at conscription. The majority among those eventually diagnosed with type 2 diabetes were recruited from the large group of men with normal BMI when young.

For the vast majority of cases, age of onset was found in the NDR (*n* = 60,307). For the remaining 3650 lacking information on year of diabetes diagnosis, registration in the NPR was used. For sensitivity analysis, an additional 5362 individuals were identified as having type 2 diabetes through the NPR. **Supplementary Figures S2** and **S3** show analyses including these individuals, with similar results as in the main analyses. **Supplementary Figure S4** shows analyses stratified by muscle strength, cardiorespiratory fitness and IQ with BMI as a continuous variable, where lower IQ, muscle strength, and cardiorespiratory fitness seems to be associated with a higher risk of diabetes given adolescent BMI.

## Discussion

Using data from a combination of Swedish registers, we investigated the association between adolescent BMI and the risk of type 2 diabetes across a wide BMI range, including over 6500 severely obese adolescent men (BMI ≥35 kg/m^2^). These young men displayed a 15-fold risk that of lean men (BMI 18·5 to <20 kg/m^2^), with a risk of type 2 diabetes before the age of 60 estimated at nearly 40%. The risk of type 2 diabetes was over twice that of lean men, already at high normal BMI levels. Among men who developed diabetes in adulthood, those who were severely obese in adolescence developed diabetes at a younger age, with poorer metabolic control at onset and with early evidence of renal stress, compared with normal weight men.

Men who were conscripted in later, as compared with earlier years, had higher BMI, and a higher proportion were obese, as reported previously,^26^ reflecting the increase of obesity in society. Shorter mean follow-up time for those with high conscription BMI may have affected the mean age of diabetes onset, estimated through age at NDR registration, which was lower in this group. This difference in follow-up time could attenuate the impact of BMI on diabetes risk in the highest BMI categories. In contrast, for young people, less frequently in contact with health care, the probability of asymptomatic diabetes being discovered is potentially lower, leading to a longer duration of undiagnosed diabetes. Between 1975 and 2016 the global prevalence of obesity in youths increased from 0·7 to 5·6% in girls and 0·9 to 7·8% in boys.^27^ In Sweden, the prevalence of obesity in youths showed a near 5-fold increase between 1969–1974 and 2000–2005, from 0·8 to 3·8%.^26^ Our findings support an expected rise in type 2 diabetes, while also indicating onset at a younger age, resulting in higher risk of complications, and more life years lost from diabetes.^18^

A meta-analysis on the association between overweight, obesity and risk of type 2 diabetes showed that the average relative risk is approximately 3-fold in overweight subjects and 7-fold in obese subjects compared to subjects with normal BMI. This meta-analysis included studies on both sexes within a broad age span (18–80 years) .^4^ Several studies have shown that high adolescent BMI increases risk of type 2 diabetes, although with relatively small sample sizes and varying definition of type 2 diabetes. A Danish study investigated men born in 1955 eligible for conscription at age 18–19 years, followed until the age of 55, with information on cardiovascular morbidity including type 2 diabetes acquired using a combination of patient registers and information on anti-diabetic drug prescription. The HR for type 2 diabetes was estimated at 3·1 (95% CI: 2·4–4·0) for overweight, and 8·2 (95% CI: 5·4–12·3) for obese individuals compared to normal BMI (18·5–< 25 kg/m^2^),^13^ comparable to our results. However, the combined overweight and obese category included a total of only 102 men who developed diabetes, and the effect of severe obesity, the most rapidly growing category,^26^ was not estimated. With many patients being treated in primary care^14^ or receiving lifestyle modification advice only,^20^ using information on hospitalizations and anti-diabetic drugs can lead to prevalence underestimation.

Another study by Tirosh et al. on BMI of 37,674 male Israeli conscripts, aged 17, reported HR for incident diabetes of 2·76 (95% CI 2·11–3·58) comparing the highest and lowest BMI decile, albeit reduced to non-significance after adjustment for adult BMI.^8^ In our study, we found that BMI in adolescence was a strong predictor of type 2 diabetes with a detectable risk increase already at low normal levels. Because we lacked information on adult BMI for non-diabetic controls, we were unable to make adjustment for this, but among those who developed diabetes, weight gain from adolescence to adulthood was substantial. Adjustment for adult BMI would conceivably have decreased HRs substantially. However, given the considerable tracking of weight status from youth into middle age, elevated adolescent BMI still serves as a useful marker of future obesity.^8^

Our study also shows an association between adolescent BMI and risk for albuminuria in subjects who developed type 2 diabetes. A previous study has shown an association between duration of obesity and chronic kidney disease, attenuated but still evident when adjusting for diabetes,^28^ indicating that the increased risk of albuminuria with elevated adolescent BMI seen in our study may be generalized. Both elevated blood pressure and altered adiposity have been suggested as causal in promoting albuminuria.^29^ The perceived risk of albuminuria among subjects who were underweight at NDR registration seen in our study should be interpreted with caution due to the small number of men in this category, and a relatively high proportion of missing values.

A strength of our study is the large study population, consisting of over 1·6 million men. The ascertainment of the diagnosis relied to a major extent on a clinical diagnosis of type 2 diabetes, which we believe is a strength when comparing to earlier studies, which have been forced to rely on self-reported diagnoses,^7^ information on diabetes treatment,^5^ or patient registers.^12^^,^^13^ Expanding the analysis to include individuals not registered in the NDR but diagnosed with type 2 diabetes in the NPR did not affect the strong association between BMI and risk of type 2 diabetes.

Limitations include that BMI, particularly within the normal range, does not take into account anthropometric distribution of body fat or distinguishes between muscular or adipose tissue. Another limitation is that we had no access to BMI measurements later in life for conscripts who did not develop diabetes. Therefore, how the risk of type 2 diabetes relates to weight gain, adult weight or duration of obesity could not be investigated. 8% and 24·5% of BMI values were missing from the conscript register and the NDR, respectively. In the conscript register, missing BMI values showed no pattern by year. A possible pattern was seen regarding missing values in the NDR with a tendency for the oldest to have more missing values. This is a potential source of bias. On the other hand, including the oldest men with potentially somewhat worse health in middle age and later in the analyses could also affect the results as age and morbidity may affect BMI.

Coverage of diabetes diagnoses in the NDR has been estimated at 90%,^16^^,^^20^^,^^21^ leaving an estimated 10% of missing diabetes diagnoses. This is a potential source of bias where year of birth, socioeconomic status and country of origin are all factors which could potentially be associated with a higher risk of missing diagnoses. However – we believe that the generally high coverage of the NDR means that these are relatively few and should not significantly affect the strong association seen between adolescent BMI and incident diabetes.

Finally, a significant limitation is that the study population is restricted to young men and the results may not be applicable to women. Previous studies using data from the Nurses’ Health Study have shown association between weight gain from late adolescence into adulthood,^30^ as well as self-reported weight at 18 years of age and risk of type 2 diabetes – although the latter was reduced to non-significance after adjustment for current BMI.^31^

In conclusion, our results show a strong correlation between BMI in adolescence and risk of subsequent type 2 diabetes. This was not limited to those overweight in adolescence, but apparent already at normal BMI levels, likely mediated by adult weight gain. Overweight and obese men subsequently diagnosed with type 2 diabetes had far earlier onset, poorer metabolic control, higher blood pressure and more albuminuria compared to lean or normal weight men, accounting for age and diabetes duration. Our findings indicate that the current high, and increasing, prevalence of obesity and severe obesity in the young will lead to a rise in type 2 diabetes, diagnosed in younger patients, with poorer metabolic control at onset, and greater risk of complications, accentuating the need of early preventative efforts against childhood and adolescent obesity.

## Funding

This work was supported by grants from the Swedish state under the agreement concerning research and education of doctors (ALFGBG-717,211, ALFGBG-881381); the Swedish Heart and Lung Foundation [Grant No. 2018-0366], the Swedish Diabetes Foundation; and the Swedish Research Council (2018-02527, VRREG 2019-00193).

## Contributors

AR, JE and KA conceptualised the study. JE performed the statistical analyses. KA conducted literature search and wrote the original draft of the report. MA and JE verified the underlying data. All authors provided critical review, commentary and contributed to the revision of the article manuscript. All authors had access to the underlying data.

### Data sharing

Data are available from the sources stated in the paper on request to the data providers, fulfilling legal and regulatory requirements and with permission from the Swedish Ethical Review Authority.

## Declaration of interests

NS has consulted for Affimune, Amgen, Astrazeneca, Boehringer Ingelheim, Eli-Lilly, Novartis, Novo Nordisk, Pfizer and Sanofi and received grant support from Boehringer Ingelheim. MLind has consulted for Astra Zeneca, Boehringer Ingelheim, DexCom, Eli-Lilly, MSD and Novo Nordisk, and received research grants from Eli-Lilly and Novo Nordisk, all outside the submitted work. Remaining authors declared no potential conflicts of interest with respect to the research, authorship, and/or publication of this article.

## Appendix Supplementary materials

Image, application 1

Image, application 2
