# Supplementary material for: Body mass index in adolescence, risk of type 2 diabetes and associated complications: A nationwide cohort study of men
Source: eClinicalMedicine. 2022 Mar 21;46:101356. doi: 10.1016/j.eclinm.2022.101356 (PMC8938860; doi:10.1016/j.eclinm.2022.101356)
Supplement: Supplementary file 1 [file mmc1.docx]

# Caption for supplementary material - Body mass index in adolescence, risk of type 2 diabetes and associated complications

**Table S1 -** Description of the definition of type 2 diabetes and year of onset.

**Table S2 -** Change in body mass index at the time of registration in NDR, stratified by body mass index (kg/m2) at conscription

**Figure S1 -** Flow-chart for the final cohort at the time of conscription

**Figure S2 -** Kaplan Meier Survival curve for incident type 2 diabetes stratified by body mass index at conscription, including additional cases based on registered diagnosis in the national patient register with diabetes code according to ICD 8, ICD 9 or ICD 10.

**Figure S3 -** Hazard ratio and incidence rates for the risk of incident type 2 diabetes during follow- up, stratified by body mass index at conscription, including additional cases based on registration in the national patient register with diabetes code according to ICD 8, ICD 9 or ICD 10

**Figure S4 -** Hazard ratio for the risk of incident type 2 diabetes during follow- up, stratified by level of muscle strength, cardiovascular fitness (FYSA) and IQ

**Figure S5 -** Hazard ratio and incidence rates for the risk of incident type 2 diabetes during follow- up, stratified by body mass index at conscription, further adjusted for parental education
