# Supplementary material for: Body mass index in adolescence, risk of type 2 diabetes and associated complications: A nationwide cohort study of men
Source: eClinicalMedicine. 2022 Mar 21;46:101356. doi: 10.1016/j.eclinm.2022.101356 (PMC8938860; doi:10.1016/j.eclinm.2022.101356)
Supplement: Supplementary file 2 [file mmc2.docx]

**Appendix**

**Supplementary table S1 – Description of the definition of type 2 diabetes and year of onset.**

| Follow up years (years) (median) | 29.0 |
| --- | --- |
| Definition of type 2 diabetes based on registration in the NDR | |
| - Clinically defined type 2 diabetes in the NDR with a set year of onset (n) | 60,307 |
| - Clinically defined type 2 diabetes in the NDR (unknown year of onset, known first diagnosis in the national patient register) (n) | 1,823 |
| - Clinically defined type 2 diabetes in NDR (unknown age of onset, unknown first diagnosis in the national patient register, known first registration date in NDR) (n) | 1,827 |
| Definition through registration in the national patient register (used for supplementary analyses) | |
| Definition based on ICD-10 | |
| - Type 2 diabetes with prevalent ICD-10 code E11 (n) | 2,938 |
| Definition based on ICD-8, ICD-9, ICD-10 | |
| - Type 2 diabetes with prevalent diabetes diagnosis not containing E11 but with an age of ≥30 years at the date of diagnosis (n). ICD-10 code E10 (type 1 diabetes) censored. | 2,424 |

Data as (n) unless otherwise specified. Definitions based on NDR used in main analyses where year of onset was defined in hierarchical order as 1: registered year of debut in the NDR, 2: registered in the national patient register with ICD diabetes code, 3: via first registration date in the NDR.

Diabetes not registered in the NDR but solely identified through registration in the national patient register with diabetes ICD codes used only in supplementary analysis.

| Supplementary table S2 – Change in body mass index at the time of registration in NDR, stratified by body mass index (kg/m2) at conscription | | | |
| --- | --- | --- | --- |
|  | **n** | **Change in body mass index (kg/m2)** | **Change in body mass index (%)** |
| Overall | 63957 | 8.6 (4.9) | 38.6 (22.8) |
| *BMI-group* |  |  |  |
| <18.5 | 3913 | 9.7 (3.9) | 54.8 (21.9) |
| 18.5-<25 | 42697 | 9.1 (4.5) | 42.4 (21.4) |
| 25-<30 | 12731 | 7.8 (5.3) | 29.1 (20.0) |
| 30-<35 | 3687 | 5.9 (6.1) | 18.7 (19.3) |
| 35 or above | 929 | 3.9 (6.9) | 10.4 (18.4) |

Data as mean (sd).

Abbreviations: body mass index (BMI).

Exclude individuals with age <18 years or >24 years

Individuals, n = 1,874,651

60,113 (3.21 %)

Individuals, n = 1,814,538

Exclude individuals with missing BMI

149,099 (7.95 %)

Exclude individuals with an age of onset earlier, or at the same age as the age of conscription

Individuals, n = 1,665,439

1,301 (0.069 %)

Exclude individuals with negative follow-up time

Individuals, n = 1,664,138

170 (0.009 %)

Exclude individuals with a measured height of < 140 cm, >210 cm or a weight < 50 kg or >140 kg

Individuals, n = 1,663,968

9,485 (0.506 %)

Exclude patients with previous registered alcohol abuse, substance abuse, cancer, Grown-Up Congenital Heart Disease, acute myocardial infarction, stroke, coronary heart disease, heart failure or deep venous thrombosis

Individuals, n = 1,654,483

6,657 (0.355 %)

Individuals, n = 1,647,826

Final cohort

**Supplementary Figure S1**

Flow-chart for the final cohort at the time of conscription

Data as n (% of the original cohort comprising of 1,874,651 men)


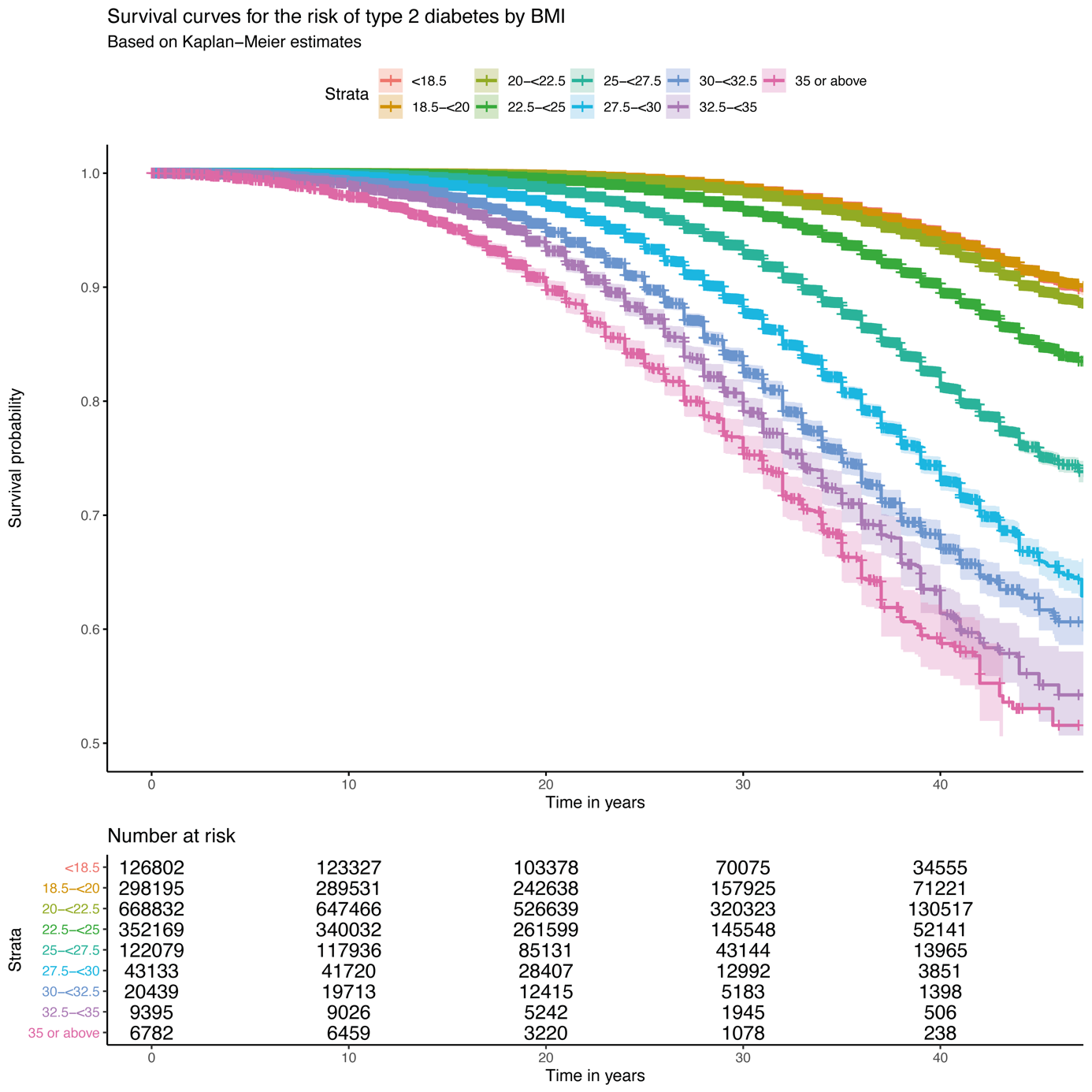
**Supplementary Figure S2 – Kaplan Meier Survival curve for incident type 2 diabetes stratified by body mass index at conscription, including additional cases based on registered diagnosis in the national patient register with diabetes code according to ICD 8, ICD 9 or ICD 10.**

Abbreviations: body mass index (BMI)


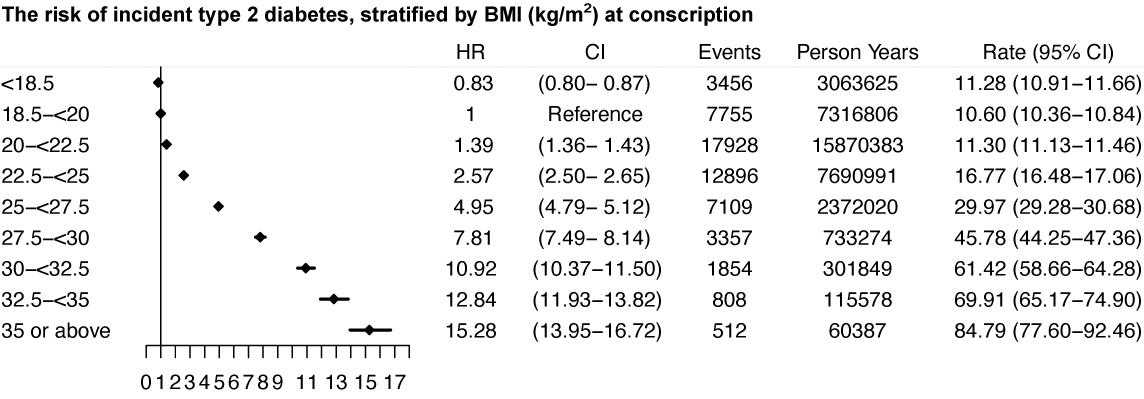


**Supplementary Figure S3 – Hazard ratio and incidence rates for the risk of incident type 2 diabetes during follow- up, stratified by body mass index at conscription, including additional cases based on registration in the national patient register with diabetes code according to ICD 8, ICD 9 or ICD 10**

Analyses based on Cox regression, adjusted for age, year of conscription and center, cardiorespiratory fitness and muscle strength. Rate as events per 10,000 person years. Abbreviations: body mass index (BMI).


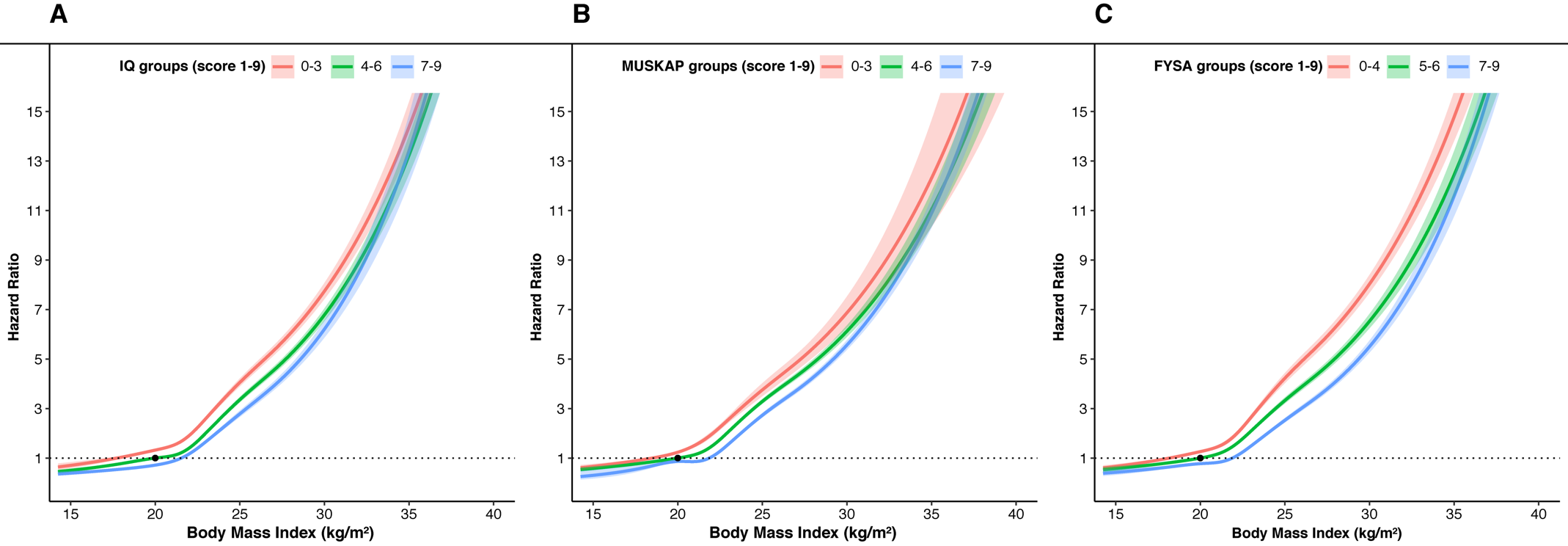


**Supplementary Figure S4 – Hazard ratio for the risk of incident type 2 diabetes during follow- up, stratified by level of muscle strength, cardiorespiratory fitness (FYSA) and IQ**

Analyses were based on Cox regression adjusted for age, year of conscription, hypertension and center and additionally cardiorespiratory fitness, muscle strength, IQ where relevant. Panel A, hazard ratio for the risk of type 2 diabetes modelled as an interaction between body mass index at conscription and IQ group; Panel B, hazard ratio for the risk of type 2 diabetes modelled as an interaction between body mass index at conscription and MUSKAP group; Panel C, hazard ratio for the risk of type 2 diabetes modelled as an interaction between body mass index at conscription and FYSA group. MUSKAP, muscle capacity, FYSA, physical capacity.


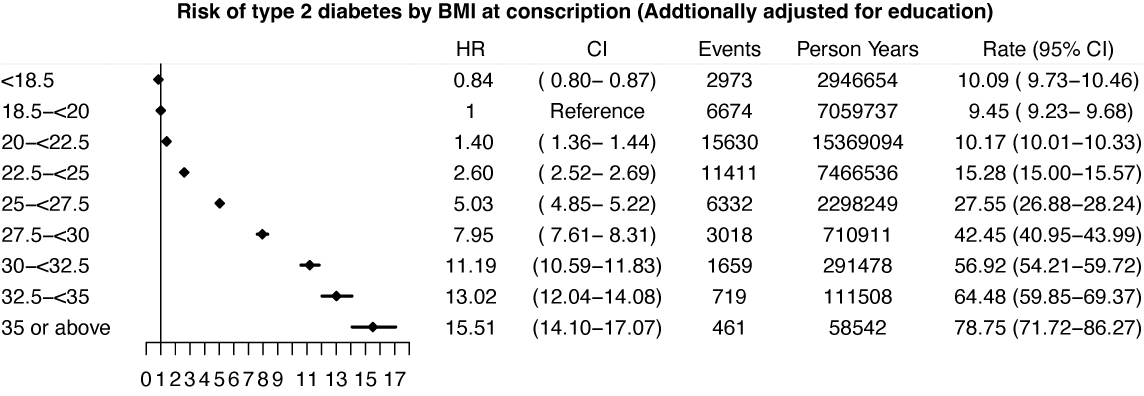


**Supplementary Figure S5 – Hazard ratio and incidence rates for the risk of incident type 2 diabetes during follow- up, stratified by body mass index at conscription**

Analyses were based on Cox regression. Adjusted for age, year of conscription and center, cardiorespiratory fitness, muscle strength and parental education. Rate as events per 10,000 person years. Abbreviations: body mass index (BMI).
